# Inkjet Printing on a New Flexible Ceramic Substrate for Internet of Things (IoT) Applications

**DOI:** 10.3390/mi11090841

**Published:** 2020-09-08

**Authors:** Sharadindu Gopal Kirtania, Manjurul Ahsan Riheen, Sun Ung Kim, Karthik Sekhar, Anna Wisniewska, Praveen Kumar Sekhar

**Affiliations:** 1 School of Engineering and Computer Science, Washington State University, Vancouver, WA 98686, USA; sharadindu.kirtania@wsu.edu (S.G.K.); m.riheen@wsu.edu (M.A.R.); sunung.kim@wsu.edu (S.U.K.); anna.wisniewska@wsu.edu (A.W.); 2 Department of ECE, Faculty of Engineering and Technology, SRM Institute of Science and Technology, Vadapalani Campus, No.1, Jawaharlal Nehru Road, Vadapalani, Chennai, TN 600026, India; karthiks1@srmist.edu.in

**Keywords:** printing parameters, sintering, curing, electrical resistivity analysis of silver nanoparticles, IoT, bending analysis, analysis of variance (ANOVA)

## Abstract

In this article, the optimization of printing properties on a new, flexible ceramic substrate is reported for sensing and antenna applications encompassing internet of things (IoT) devices. E-Strate^®^ is a commercially available, non-rigid, thin ceramic substrate for implementing in room temperature and high-temperature devices. In this substrate, the printing parameters like drop spacing, number of printed layers, sintering temperature, and sintering time were varied to ensure an electrically conductive and repeatable pattern. The test patterns were printed using silver nanoparticle ink and a Dimatix 2831 inkjet printer. Electrical conductivity, high-temperature tolerance, bending, and adhesion were investigated on the printed samples. The three-factor factorial design analysis showed that the number of printed layers, sintering temperature, sintering time, and their interactions were significant factors affecting electrical conductivity. The optimum printing parameters for the thin E-Strate^®^ substrate were found to be 20 μm drop spacing, three layers of printing, and 300 °C sintering temperature for 30 min. The high-temperature tolerance test indicated a stable pattern without any electrical degradation. Repetitive bending, adhesion test, and ASTM tape tests showed adequate mechanical stability of the pattern. These results will provide insight for investigators interested in fabricating new IoT devices.

## 1. Introduction

There is an unmet need for integrated, inexpensive, and conformal devices with a smaller footprint in the era of the internet of things (IoT) [1,2,3]. In particular, the IoT framework in nuclear power plants [4], industrial compressors [5], automotive industry [6], oil refineries [7], aerospace propulsion systems [8], etc. requires wireless sensing systems to monitor physical and chemical parameters accurately. To improve efficiency, safety, and diagnostics, hundreds of these wireless sensors are required to fit in critical areas, planar or non-planar in nature. In addition, federal agencies in the U.S [9] and industry are looking for an integrated, high-temperature operable, and flexible wireless sensor system solution [10]. In this context, the need for a flexible substrate conducive to room temperature and high-temperature operation becomes relevant.

Commonly used substrates for flexible devices such as sensors and antenna include different types of polymer and paper substrates [11]. While the paper and polymer substrates are suitable for room temperature device operation [12], high-temperature operation require ceramic substrates. E-strate^®^ is a commercially available thin, flexible ceramic substrate that enables device operation at room temperature and high temperature (up to 1000 °C).

E-strate^®^ is made of 3 mol% partially yttria-stabilized zirconia (3YSZ) [13]. Due to its high dielectric constant, low thermal conductivity, superior mechanical properties, and tolerance to high temperatures, E-strate^®^ has enabled radio frequency, photonics, chemical sensing, and renewable energy applications [14,15,16,17]. Various metallization techniques have been investigated on this substrate for device fabrication, while inkjet printing for device fabrication has been relatively unexplored.

Inkjet printing technology is a simple yet effective method to fabricate sensors, antennas, integrated circuits, batteries, displays, Radio-frequency identification (RFID) tags, thin-film transistors, and many other devices [18]. Fabricating devices using inkjet printing offers a precise definition of intricate patterns on the substrate. Typically, three types of ink are used in inkjet printing. They are metal nanoparticles (NPs) like silver (Ag), copper (Cu), or gold (Au), metal-organic decomposition (MOD) ink [19], and conductive polymers [20]. Among metal NP inks, silver has the lowest bulk resistivity, $1.59\times 10^{-8}$ Ω-m [21]. The printed pattern of Ag NPs on a substrate needs sintering for decomposing the protective agents from the surfaces of Ag NPs. Sintering ensures direct physical contact between Ag NPs and sets up a dense and conductive system throughout the printed pattern [22]. Hence, the sintering temperature and sintering time need to be carefully chosen.

Lower sintering temperature fails to yield a dense and highly conductive printed feature while a higher value degrades the pattern. Further, appropriate sintering time is critical for optimum conductivity of the printed pattern [23]. Optimization of the printer parameters for the selected ink and substrate combination is mandatory to guarantee high printing quality, as inks with varying viscosity and surface tension interact differently with various substrates. The authors have prior experience in optimizing printing properties on different substrates [24,25]. A fundamental requirement for robust device implementation is the device integrity at room temperature and high temperature in addition to flat and bent conditions. Hence, a reliable inkjet printing process is essential for large scale device fabrication.

In this context, the optimum printing parameters on thin E-strate^®^ are determined in order to obtain a highly conductive and mechanically stable printed pattern, a critical requirement of flexible devices. The printing parameters such as drop spacing, the number of printed layers, sintering temperature, and sintering duration were investigated. The electrical conductivity of the printed samples was inspected to ensure the quality of printing. To assess flexibility and pattern durability, the authors performed bending and adhesion tests.

## 2. Experimental

### 2.1. Materials

Commercially available Ag NP ink was purchased from Novacentrix^®^ (Model: Metalon-JS-B25HV). This is a water-based Ag ink with 25 wt% Ag content with viscosity is 8–10 cp and surface tension 30–32 dyne/cm. The relative density of the ink is 1.2–1.4. The z-average particle size for this ink is 60–80 nm. The thin E-strate^®^ was purchased from ENrG Inc. The substrate thickness is 40 μm, and the dimension of the substrate is 27 mm × 47 mm.

### 2.2. Printing of Test Pattern

**Figure 1:**
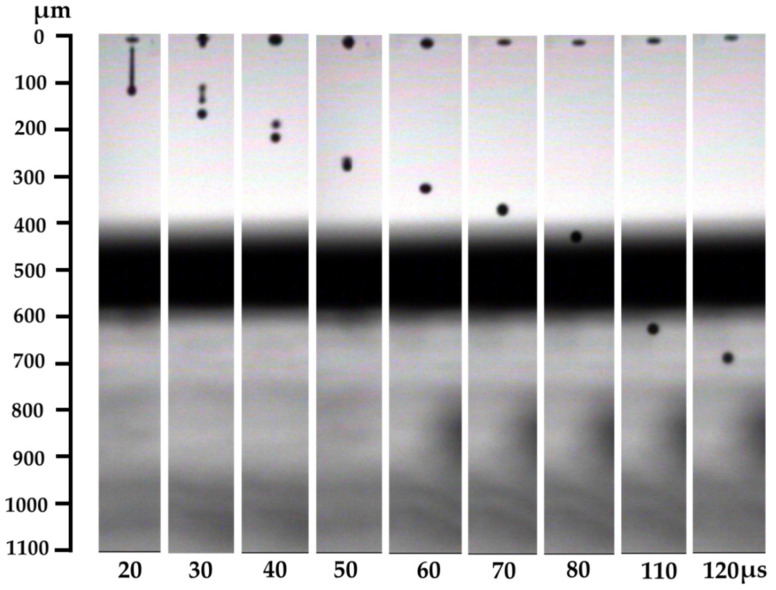
Drop formation of ink from a single nozzle in different time frame. Satellite droplets were formed at 30 µs and retracted after 50 µs. (For 90 and 100 µs, the droplet was in the shadowed part, which was not given in the figure).

A Fujifilm Dimatix Materials Printer (DMP-2831) was used to print the pattern on thin E-strate^®^. 10 pL cartridges were used for the experiment. The primary objective of the optimization is to create a consistent and straight away droplet at a velocity within the range of 5.5–6 m/s from the selected nozzles so that the jetted drops attach well to the thin E-strate^®^ surface. The droplets of ink were inspected using the drop watcher of Drop Manager Software of DMP. The jetting waveform, the jetting voltage, the jetting frequency, the cartridge temperature, the platen temperature, and the resolution of the pattern dictate the printing quality of the inkjet printer [11]. Table 1 shows the values of the parameters mentioned above. Figure 1 shows the drop formation from a single nozzle using the drop watcher camera. The jetting voltage controls the speed of the droplet firing. The printer height from the substrate affects the formation of the satellite droplets (Figure 1), printing accuracy and resolution. Initially, there were some satellite droplets within the first 30 to 50 µs. However, at a substrate height of 550 µm (around 80 µs), the droplets were seen to be steady and satellite droplets coalesced into a single droplet.

A square test pattern of dimension 15 mm × 15 mm was designed in ANSYS EM software and uploaded in the DMP. The printing parameters, such as the number of printing layers, sintering temperature, sintering time, and drop spacing (shown in Table 2) were varied, and the resistivity was recorded. The initial values of these parameters were decided based on trial and inspection.

### 2.3. Post-Processing and Resistivity Measurement

Table 1 and Table 2 show the printing specifications and experimental printing parameters, respectively.

The printed samples were sintered using a hot air gun (Model: Kendal 898$D^{+}$). Printed test patterns were very thin, and their resistances can be considered as sheet resistances, which were measured using the four-point probe method [26]. The formula below was applied to calculate the electrical resistivity (ρ):

(1) $$\rho =\left(R_{s}\times t\right)\;\Omega \text{-}\mathrm{cm}$$

where $R_{s}$ = the sheet resistance of the printed pattern (Ω/sq) and **t** = thickness of the Ag ink layer (cm). Jandal’s four-point probe was used to measure the sheet resistance. As thin E-strate^®^ is a non-porous substrate, the ink does not get absorbed on the surface. As per the material datasheet of Novacentrix^®^, high-temperature sintering (250 °C or greater) is recommended for the proper adhesion of ink to the substrate.

### 2.4. Surface Characterization

Scanning electron microscope (SEM) Quanta 3D 200i was used to measure the printed layer thickness. The test specimens were inspected using Alpha-Step^®^ D-300 Stylus Profiler to investigate the surface roughness and the uniformity of the printed pattern. The surface morphology of the different layered printed specimens was examined using the SEM.

### 2.5. High-Temperature Tolerance Test

The high-temperature tolerance of the printed pattern on thin E-strate^®^ was tested via the fabrication of a capacitive interdigitated (IDT) pattern. The IDT pattern was designed and simulated an IDT capacitor on COMSOL Multiphysics 5.4. The capacitance value was found to be 48 pF based on the simulation. Then, the IDT structure was printed on thin E-strate^®^ and sintered it at 300 °C for 30 min. The capacitance was measured using the LCR meter, Model: Amprobe LCR55A. Then, the printed capacitor was heated at 500 °C for 1 h, and the capacitance value was measured after the substrate was cooled down.

### 2.6. Flexibility and Adhesion Test

**Figure 2:**
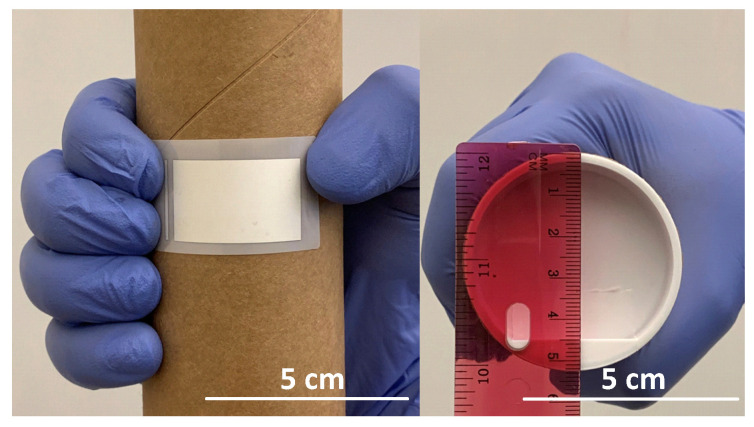
Flexibility test on a printed pattern.

The flexible substrate was subjected to repeated bending (100 cycles) to assess the mechanical and electrical integrity of the patterns as it would be the case in real-time applications. To check the flexibility of thin E-strate^®^, repetitive tensile bending, and adhesion test (100 runs) of the printed pattern were carried out. Repetitive bending test was performed by folding the printed pattern around different radii of curved structure [27,28]. The printed rectangular pattern was bent around the two cylindrical shapes of diameter 53 mm (Figure 2), and 64 mm. The ASTM standard tape test was used to check the adhesion strength of the printed pattern with the substrate.

## 3. Results and Discussion

### 3.1. Electrical Conductivity

**Figure 3:**
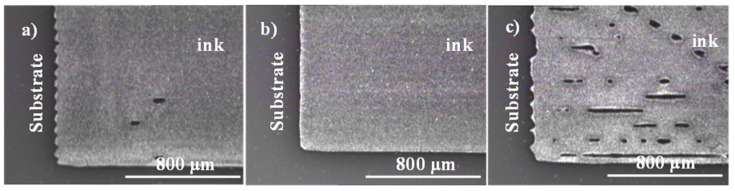
Inkjet-printed pattern: (**a**) 15 μm drop spacing, (**b**) 20 μm drop spacing, and (**c**) 25 μm drop spacing.

The pattern printed with drop spacing (DS) of 15 μm (1693 DPI), 20 μm (1270 DPI), and 25 μm (1016 DPI) were inspected using the Fiducial Camera of DMP. Figure 3 shows the surface of the pattern printed using different drop spacing. A sharp edge quality of the printed pattern at 20 μm DS was observed.

For 15 μm drop spacing, the droplets seem to become too close, which results in more ink spreading and degradation of the printed pattern edges [29]. On the other hand, a higher DS (25 μm) seems to create an irregular printed pattern with higher resistivity values.

Hence, the 20 μm DS was selected as the optimum DS. After determining the optimum DS, the correlation of the resistivity with the number of printing layers, sintering temperature, sintering time was studied. The measured resistivity as a function of sintering temperature, sintering time, and the number of printed layers is presented in Table 3. A three factorial design based statistical approach was pursued to determine the significance of sintering temperature, the number of printing layers, sintering time, and their interaction on the resistivity of the printed sample. The data for a single layer pattern is omitted from the analysis due to numerous outliers in the resistivity values (attributed to pinholes and irregular patterns). Each data point was repeated, which is indicated by ‘run 1′ and ‘run 2′ column. From Table 3, it can be inferred that resistivity values decrease with sintering time. The three-factor analysis of variance (ANOVA) is summarized in Table 4. The analysis reveals that the number of printing layers, sintering temperature, and sintering time affect resistivity significantly.

**Figure 4:**
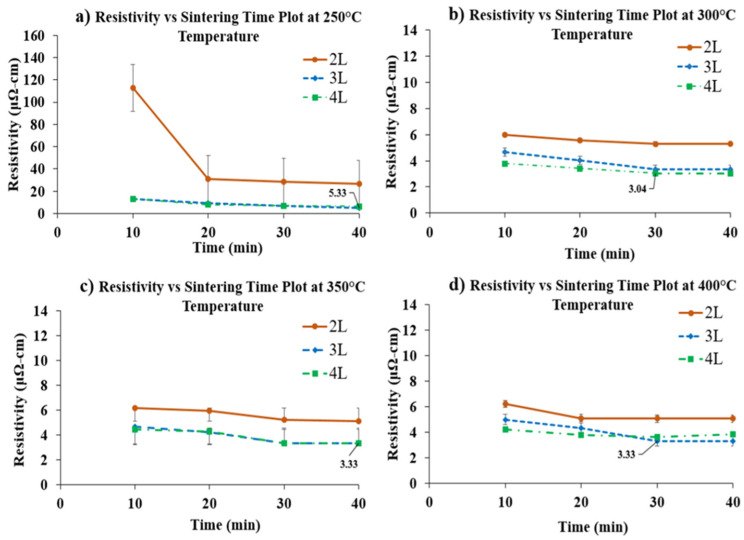
Resistivity value as a function of sintering time at different sintering temperatures: (**a**) 250 °C, (**b**) 300 °C, (**c**) 350 °C, (**d**) 400 °C.

Further, their mutual interactions seem to influence resistivity. As the F-values for printing layers and temperatures and their interaction are very high compared to other factors, they affect resistivity more significantly. Figure 4 shows an array of graphs highlighting electrical resistivity as a function of sintering time for different sintering temperatures. As the patterns require thermal sintering for significant electrical conductivity, no resistivity was measured before sintering. After minimum sintering of 10 min, the readings for resistivity were recorded. The optimum resistivity value was observed to be 3.33 μΩ-cm (desired value) for 3 L printing at 300 °C sintering temperature for 30 min of sintering. With an increase in sintering temperature from 250 °C to 400 °C, the sintering time to get the optimum value of resistivity seems to reduce. Before thermal curing, there is no physical contact between the silver nanoparticles, and the printed patterns show no conductivity.

**Figure 5:**
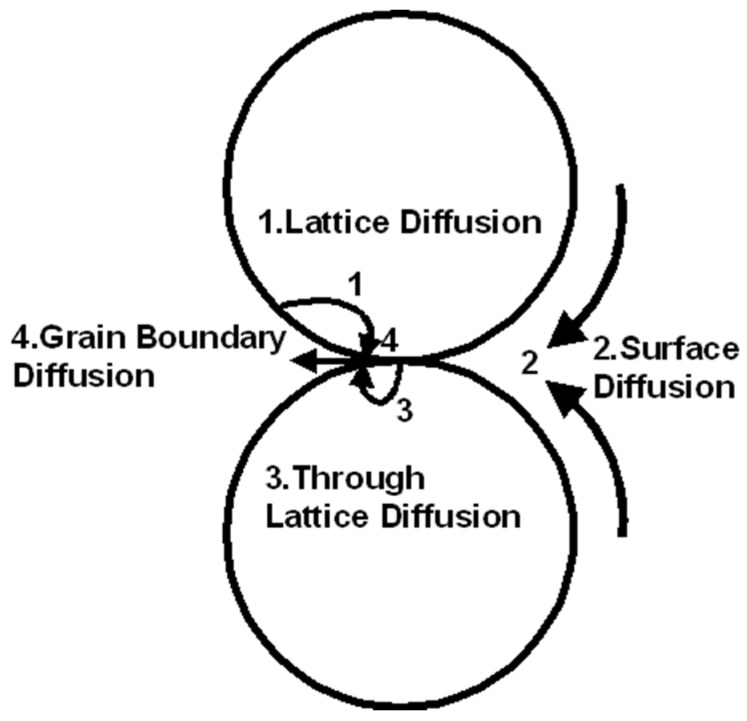
A schematic is representing various atomic diffusion paths between two contacting particles. Reprinted with permission from Ref. [31].

Heat is required to decompose the organic solvent and create metallic contact between the particles to form a conductive feature. These changes in resistance of the printed patterns arise from a material transport process based on the atomic diffusion driven by the reduction of interfacial energy [30]. For better understanding, a schematic of two interacting nanoparticles with possible diffusion paths during sintering is shown in Figure 5.

In the initial stage, the interparticle neck forms and increases the particle contact area [30]. Neck growth is the dominant factor that reduces the resistivity of sintered particles because it increases the cross-sectional contact area between the particles [32]. As the temperature is increased, the nanoparticles start forming dense agglomerates due to the fusion of particles caused by surface diffusion, grain boundary diffusion, as well as lattice diffusion [33]. The neck formation is driven by surface energy reduction due to the particles’ large surface-to-volume ratio, a process known as Ostwald ripening [34]. Ostwald ripening triggers the diffusion mechanisms, which enables more contact between the fused particles. Grain boundary diffusion allows for neck formation and neck radii increase.

Besides neck growth between several particles, percolation networks emerge due to multi-particle-related sintering [35]. These percolation networks create an electron flow path, thereby decreasing the resistivity of the pattern. The surface of Ag nanoparticles provides a sufficient path for electron flow. Finally, a coalesced densified, non-porous metallic pattern emerges on the substrate. For the sample sintered at 250 °C, the resistivity dropped rapidly during the initial stage of sintering (from 10 to 20 min) due to the earlier neck formation and faster neck growth. As the sintering process gets closer to the intermediate stage, the variation of resistivity between different times became smaller, as the rate of neck growth decreases. For temperatures above 300 °C, the rapid drop of resistivity happens around 10 min. As the sintering process gets closer to the intermediate stage, the difference in resistivity becomes smaller, since the rate of neck growth decreases during the sintering process.

**Figure 6:**
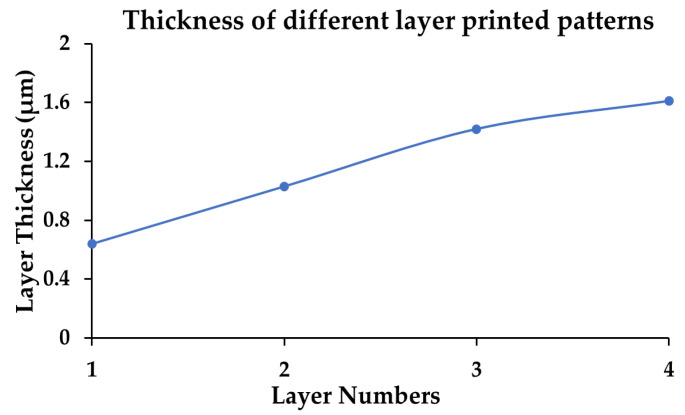
Thickness of printed pattern as a function of printed layers.

Resistivities at all temperatures fall slowly at the intermediate stage of sintering due to the relatively slow rate of neck growth and coalescence of the particles. From Figure 6, it is evident that the thickness of the pattern increases with the number of printed layers.

**Figure 7:**
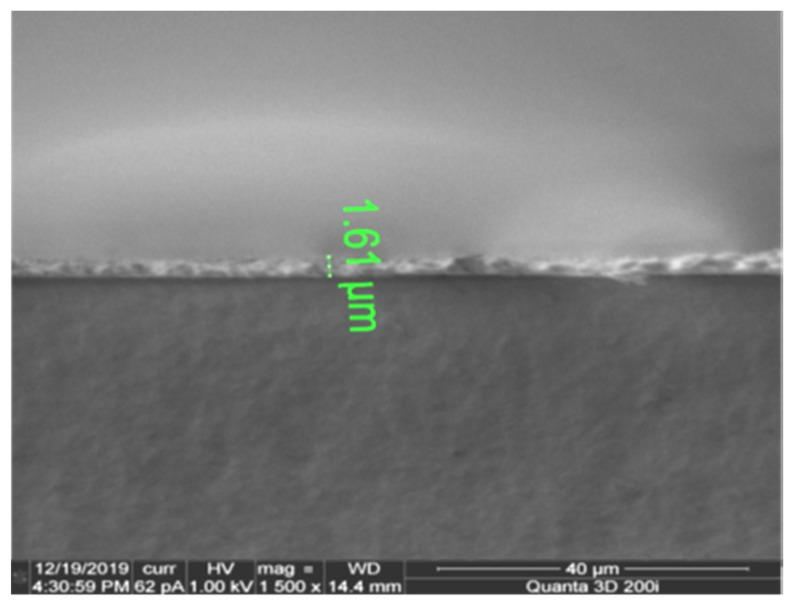
Thickness of 4-layer printed samples using SEM.

Though a thicker pattern could result in well-connected conductive pathways resulting in slightly improved conductivity (refer Table 3), the trade-off between ink consumption (cost) and electrical conductivity should not be ignored. In this context, the authors chose the conductivity of 3 L printing at 300 °C sintering temperature for 30 min as the optimum process parameter for inkjet printing. As sintering enables the nanoparticles to coalesce together, densification occurs in vertical and horizontal directions. The thickness of the printed layer did not increase linearly with an increase in the printing layer as the densification process seems non-uniform. The thickness of the pattern was measured using a cross-sectional SEM analysis. Figure 7 shows a thickness of 1.61 µm for the four-layer printed pattern. The image appears to show a good adhesion of silver nanoparticles with the substrate.

### 3.2. Surface Characterization

**Figure 8:**
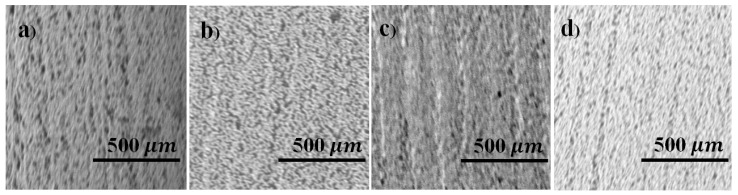
Surface morphology of the printed patterns sintered at 300 °C: (**a**) 1 L, (**b**) 2 L, (**c**) 3 L, (**d**) 4 L sintered at 300 °C.

The surface morphology of the printed pattern was studied using SEM images. Figure 8 shows the SEM images of the printed patterns (different layers) sintered at 300 °C.

**Figure 9:**
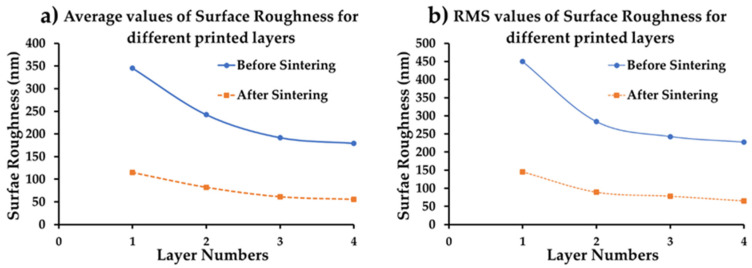
Surface roughness plot for different printed layers before and after sintering (**a**) Average value of surface roughness value, and (**b**) RMS value of surface roughness.

Figure 8a shows the single-layer printed pattern containing a lot of holes and uneven edges. The surface of the double layer printed pattern is depicted in Figure 8b. Through visual inspection, it seems that the image of a double layer printed pattern has fewer holes than the single-layered one. The number density of holes seems to decrease with an increase in the number of printed layers (calculated based on 1 µm square box and counting them). The printing quality appears to be superior for 4 L, as shown in Figure 8d. The 2D RMS surface roughness was computed using Alpha-Step^®^ D-300 Stylus Profile. Figure 9 shows the average and RMS surface roughness values as a function of the number of layers of the printed pattern before and after sintering at 300 °C. Before sintering, the roughness of the surface seems quite high, but the value seems to decrease after sintering. The surface roughness value seems to be small for a double layer printed pattern before and after sintering and continues to decrease with the number of layers. A thicker pattern is believed to allow more time for the capillary re-flow of ink, thereby creating a smooth top layer [36].

### 3.3. High-Temperature Tolerance

**Figure 10:**
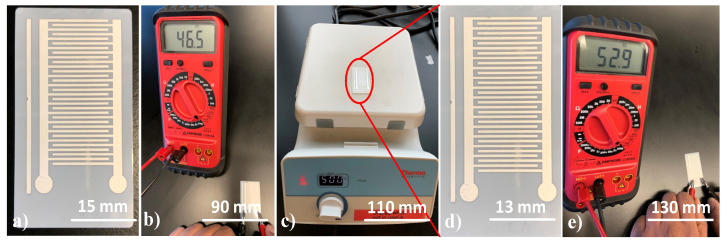
High-temperature stability test results (**a**) Interdigitated capacitive pattern after sintering, (**b**) Measuring capacitance using LCR meter, (**c**) Heating on the hotplate at 500 °C, (**d**) Capacitive pattern after one-hour heating, and (**e**) Measuring capacitance using LCR meter after heating.

The image of the printed capacitor pattern on the ceramic substrate for high temperature testing is shown in Figure 10a. After sintering the printed IDT capacitor, the measured capacitance value was found to be 46.5 pF (Figure 10b), indicating the electrical integrity of the IDT pattern. The slight difference in capacitance between the simulation (48 pF) and the experiment is attributed to imperfections in fabrication. After heating for an hour (Figure 10c), the capacitance value was found to be 52.9 pF after the substrate cooled down, indicating the absence of any degradation when subjected to high temperatures. Being a dielectric material, the dielectric constant of E-strate^®^ increases with the increase of temperature under a constant frequency [37]. The increase in capacitance after the substrate was cooled is attributed to the temperature-dependent dielectric properties of the ceramic. The temperature was limited to 500 °C due to the presence of Ag ink. Device testing at higher temperatures is possible when high-temperature tolerant inks such as Pt is used.

### 3.4. Flexibility and Adhesion Test Result

**Figure 11:**
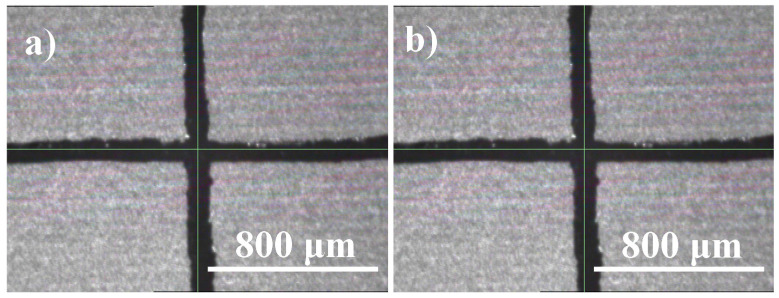
ASTM tape test: (**a**) before tape test (**b**) image after tape pull of using Fiducial camera.

The electrical resistivity was measured during and after repetitive bending. No cracks or deformation have been found after bending. A 3% change in resistivity was observed after repeated flexes (100 runs), enabling its use in flexible sensors and antenna applications. The adhesion of the Ag ink on the thin E-strate^®^ was inspected using the ASTM tape test. The surface of the substrate after the tape pull off was recorded using the Fiducial camera of the DMP-2831 printer (Figure 11). Edges of the cut were smooth with some ink detachments. Less than 5% of the test area was seen to detach due to tape pull off. Hence, the substrate is classified into a class 4B adhesion rating [38]. The sintering temperature was kept at least 250 °C for proper adhesion according to AgNP ink datasheet. A high-temperature adhesion test (using fiberglass tape) was performed at 500 °C, and the absence of significant detachment (figure not shown) was noted. Several other factors might affect adhesion, for instance, surface roughness, surface energy, and polarities of the contact surfaces, and the porosity of the substrate surfaces [39]. Sintering temperature and sintering time are other dominant factors affecting the adhesion of printed patterns on non-porous substrates [40]. The bending and adhesion tests indicate a well-sintered pattern, which is mechanically and electrically stable, conducive for implementing flexible devices for IoT applications.

## 4. Conclusions

In this article, the optimization of inkjet printing properties on a new ceramic substrate has been reported. The impact of drop spacing, number of printing layers, sintering temperature, and sintering time on the resistivity of the pattern was studied. The number of printing layers, sintering temperature, and their interaction were found to be significant factors affecting the electrical conductivity of the printed pattern. The optimum printing parameters for the thin E-strate^®^ substrate were found to be 20 μm drop spacing, three layers of printing, and 300 °C sintering temperature for 30 min. The physics of resistivity evolution during sintering has been discussed. The printed sample was found to be exhibit high-temperature tolerance with adequate flexibility and adhesion to the substrate. These results indicate the suitability of the ceramic substrate for multi-functional flexible and wearable sensors for IoT applications.

## Figures and Tables

**Table 1:** Printing specifications.

Parameter	Value
Jetting Voltage (V)	15.4
Jetting Frequency (kHz)	4.4
Drop Size (pL)	10
Printing Print Height (μm)	550
Cartridge Temperature (°C)	31
Platen Temperature (°C)	50

**Table 2:** Experimental printing parameters.

Parameter	Value	Unit
Drop Spacing	15, 20, 25	μm
Printed Layer Numbers	1, 2, 3, 4	
Sintering Temperature	250, 300, 350, 400	°C
Sintering Time	10, 20, 30, 40	min

**Table 3:** Measured resistivity as a function of sintering time, sintering temperature, and the number of printed layers.

Sintering Temperature (°C) (A)	Resistivity (μΩ-cm)						
	Number of Layers (B)	2 L		3 L		4 L	
	Sintering Time (C)	run-1	run-2	run-1	run-2	run-1	run-2
250	10	113.52	112.24	12.65	13.59	13.59	12.65
	20	30.92	30.57	8.66	9.47	7.55	8.66
	30	28.02	29.01	7.33	6.66	6.8	7.04
	40	26.57	26.57	5.33	5.33	6.04	6.66
300	10	5.8	6.16	4.66	4.68	3.78	3.78
	20	5.31	5.81	4	4.1	3.4	3.4
	30	5.31	5.31	3.33	3.33	3.02	3.05
	40	5.31	5.31	3.33	3.33	3.02	3.05
350	10	6.16	6.16	4.75	4.58	4.29	4.55
	20	5.81	6.12	4.25	4.2	4.21	4.33
	30	5.21	5.2	3.33	3.33	3.35	3.33
	40	5.12	5.15	3.33	3.33	3.33	3.33
400	10	5.8	6.66	4.66	5.33	4.15	4.33
	20	5.07	5.15	4	4.66	3.78	3.78
	30	5.07	5.07	3.33	3.33	3.78	3.53
	40	5.07	5.07	3.33	3.33	3.78	3.87

**Table 4:** Analysis of Variance (ANOVA) chart.

Source of Variation	Sum of Squares (SS)	Degrees of Freedom (DF)	Mean Square (MS)	F_critical_	F	*p*-Values
Sintering Temperature (A)	5796.77	3	1932.26	2.80	19267.78	*p* << 0.05
Number of printing layers (B)	2846.73	2	1423.36	3.19	14,193.29	*p* << 0.05
Sintering Time (C)	1372.86	3	457.62	2.80	4563.21	*p* << 0.05
AB	6190.36	6	1031.73	2.29	10,288.02	*p* << 0.05
CA	3978.44	9	442.05	2.08	4407.96	*p* << 0.05
BC	1525.08	6	254.18	2.29	2534.59	*p* << 0.05
ABC	3935.35	18	218.63	1.82	2180.11	*p* << 0.05
Error	4.81	48	0.10			
Total	25,650.40	95				
